# GLOBAL ENDOCRINOLOGY: Global perspectives in endocrinology: coverage of iodized salt programs and iodine status in 2020

**DOI:** 10.1530/EJE-21-0171

**Published:** 2021-05-14

**Authors:** Michael B Zimmermann, Maria Andersson

**Affiliations:** 1 Department of Health Sciences and Technology, Human Nutrition Laboratory, ETH Zürich, Zürich, Switzerland; 2 Iodine Global Network, Ottawa, Ontario, Canada; 3 Nutrition Research Unit, University Children’s Hospital Zürich, Zürich, Switzerland

## Abstract

Iodine deficiency has multiple adverse effects on growth and development. Diets in many countries cannot provide adequate iodine without iodine fortification of salt. In 2020, 124 countries have legislation for mandatory salt iodization and 21 have legislation allowing voluntary iodization. As a result, 88% of the global population uses iodized salt. For population surveys, the urinary iodine concentration (UIC) should be measured and expressed as the median, in μg/L. The quality of available survey data is high: UIC surveys have been done in 152 out of 194 countries in the past 15 years; in 132 countries, the studies were nationally representative. The number of countries with adequate iodine intake has nearly doubled from 67 in 2003 to 118 in 2020. However, 21 countries remain deficient, while 13 countries have excessive intakes, either due to excess groundwater iodine, or over-iodized salt. Iodine programs are reaching the poorest of the poor: of the 15 poorest countries in the world, 10 are iodine sufficient and only 3 (Burundi, Mozambique and Madagascar) remain mild-to-moderately deficient. Nigeria and India have unstable food systems and millions of malnourished children, but both are iodine-sufficient and population coverage with iodized salt is a remarkable 93% in both. Once entrenched, iodine programs are often surprisingly durable even during national crises, for example, war-torn Afghanistan and Yemen are iodine-sufficient. However, the equity of iodized salt programs within countries remains an important issue. In summary, continued support of iodine programs is needed to sustain these remarkable global achievements, and to reach the remaining iodine-deficient countries.

## Invited Author’s profile


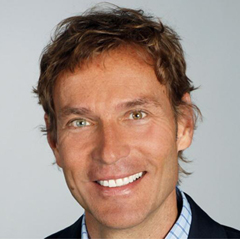


**Michael Zimmermann** received his MD from Vanderbilt University School of Medicine and did his postgraduate medical training at the University of California in San Francisco, CA, USA. He is currently a professor of Human Nutrition at the Swiss Federal Institute of Technology (ETH) in Zurich, Switzerland, and an adjunct professor in Endocrinology and Diabetes at the University of Zurich Hospital, Switzerland. He is currently chair of the Iodine Global Network, a non-profit, non-governmental organization for the sustainable elimination of iodine deficiency worldwide. His main research interests are iron and iodine nutrition.

## Introduction

Iodine deficiency has multiple adverse effects on growth and development in humans, collectively termed as the 'iodine deficiency disorders' (IDDs) (1, 2). They result from inadequate thyroid hormone production due to a lack of sufficient dietary iodine. The daily recommended nutrient intakes for iodine are: in children 0–5 years, 90 μg; in children 6–12 years, 120 μg; in adults, 150 μg; and in pregnancy and lactation, 250 μg (2). Most foods have low amounts of iodine, and diets in many countries cannot provide adequate iodine without iodine fortification of salt (1). The WHO's first estimate of the global prevalence of goiter in 1960 suggested that 20% to 60% of the world's population was affected, with most of the burden in low- and middle-income countries (3). Subsequently, the International Child Development Steering Group highlighted iodine deficiency as one of four key global risk factors for impaired child development where the need for intervention was urgent (4). Programs against IDDs are appealing for national governments because the human, economic, and social consequences can be averted by salt iodization, a low-cost and sustainable intervention. Since 1990, the elimination of IDD has been a component of many national nutrition strategies (2, 5).

### Global reach of iodized salt programs

Salt iodization has been introduced in many countries around the world as a sustainable strategy to improve the population level iodine intake and prevent IDD. Between 1942 and 2020, 123 countries introduced mandatory legislation on salt iodization. In 2021, 124 countries have legislation for mandatory salt iodization and at least 21 countries have legislation allowing voluntary salt iodization (6). Mandatory legislation is considered the most reliable approach to ensure effective salt iodization, but voluntary salt iodization can also be effective. The majority of the countries with voluntary fortification are iodine-sufficient at the national level (6, 7). Salt is considered adequately iodized when the fortification level is 15–40 ppm iodine in salt (8, 9).

**Figure 1:**
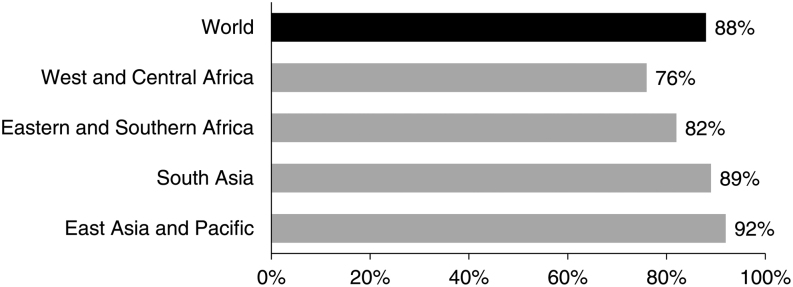
Percentage of households consuming salt with any iodine, by UNICEF region 2013–2018. Adapted with permission from (10).

The reach of current iodized salt programs is remarkable. UNICEF estimates that, based on data collected during the period 2013–2018, in 2018, 88% of the global population used iodized salt (10). As shown in Fig. 1, South Asia and East Asia and the Pacific had the highest household coverage with iodized salt of 89% and 92%, respectively. Western and Central Africa had the lowest coverage with iodized salt, but still, over three in four households in the region had access to iodized salt.

Despite this rapid progress, there remain concerns. First, UNICEF states there are insufficient recent data available to generate estimates of iodized salt coverage for Central Asia, Latin America and the Caribbean, and the Middle East and North Africa (10). The Global Fortification Data Exchange reports that only 89 of the 145 countries with legislation on salt iodization have gathered recent data on population coverage (6). Secondly, the UNICEF data refer to salt with any amount of iodine (>0 ppm) but the ultimate goal of iodized salt programs is to achieve ‘sufficiently’ iodized salt, that is, in the range of 15–40 ppm (8, 9). Finally, UNICEF estimates that nearly 1 billion people that may benefit from iodized salt still do not have access (10).

### Monitoring iodine status in populations

The impact of salt iodization on population iodine intake is monitored using biomarkers of iodine status. Several methods are recommended for the assessment of iodine nutrition in populations: urinary iodine concentration (UIC), thyroglobulin concentration in blood and goiter rate (1). These indicators are complementary, in that UIC is a sensitive indicator of recent iodine intake (days), thyroglobulin indicates increased thyroid activity and shows an intermediate response (weeks to months), whereas the goiter rate reflects long-term moderate-to-severe iodine deficiency (months to years) (2). Because >90% of ingested iodine appears in the urine in the following 24 to 48 h, UIC is an excellent indicator of recent iodine intake (11). UIC can be expressed as a concentration (μg/L), in relation to urinary creatinine excretion (μg iodine/g creatinine), or as 24-h urinary excretion (μg/day). For population surveys or cross-sectional studies, it is recommended that UIC be measured in spot urine specimens from a representative sample of the target group, and expressed as the median, in μg/L (Table 1) (2). Variations in hydration among individuals generally even out if the sample size is large enough (11, 12).

**Table 1:** Epidemiologic criteria for assessment of iodine nutrition in a population based on median or range of median urinary iodine concentrations (2).

Urinary iodine, μg/L	Iodine intake	Iodine nutrition
**School-aged children**		
<20	Insufficient	Severe iodine deficiency
20–49	Insufficient	Moderate iodine deficiency
50–99	Insufficient	Mild iodine deficiency
100–299^a^	Adequate	Optimum
>300	Excessive	Risk of adverse health consequences*
**Pregnant women**		
<150	Insufficient	
150–249	Adequate	
250–499	More than adequate	
≥500^b^	Excessive	
**Lactating women**^c^		
<100	Insufficient	
≥100	Adequate	
**Children less than 2 years of age**		
<100	Insufficient	
≥100	Adequate	

^a^Adapted with permission from (13); ^b^The term excessive means in excess of the amount needed to prevent and control iodine deficiency; ^c^In lactating women, the numbers for median urinary iodine concentration are lower than the iodine requirements, because of the iodine excreted in breast milk (15); *consequences include iodine-induced hyperthyroidism, autoimmune thyroid disease.

Although the mUIC is a good indicator of iodine status in populations, its value for assessing individual status is limited by high day-to-day variability in iodine intakes (11). In adults, intra-individual variation in UIC is high for both 24-h collections and spot samples, so that urine samples from ≈10 different days are needed to assess individual iodine status with 20% precision (12). The distribution of UICs in population studies is often misinterpreted. A common mistake is to assume that all subjects with a spot UIC < 100 μg/L are iodine-deficient (9). But because individual iodine intakes are highly variable from day-to-day, on any given day, some individuals will inevitably have a low UIC, despite average daily intakes that are adequate to maintain thyroidal iodine stores (12). Recent data suggest that the WHO mUIC categories (Table 1) as ‘adequate’ and ‘more than adequate’ iodine intake recommended in children can be combined into a single category (100–299 µg/L) to denote adequate iodine nutrition (13). Although the mUIC does not provide direct information on thyroid function, a low value suggests that a population is at a higher risk of developing thyroid disorders (14).

Pregnant and lactating women have high iodine requirements and may be at a higher risk of low iodine intake. Targeted monitoring of iodine status in these vulnerable groups is important, particularly in countries where the general population has borderline adequate iodine intakes or mild iodine deficiency. In pregnant women, iodine status is monitored using UIC and thyroglobulin (2, 15, 16), whereas iodine status in lactating women should be monitored by measuring the UIC and iodine concentration in breast milk (2, 15, 17).

### The Global Scorecard of iodine nutrition

The Iodine Global Network (IGN) compiles data from UIC studies conducted throughout the world and continually monitors global iodine status (7). The IGN Scorecard presents the most recent UIC data for 194 WHO Member States, plus Liechtenstein and Palestine.

In population monitoring of iodine status using UIC, the WHO recommends that school-age children (SAC) serve as a proxy for the general population (2). Therefore, in the IGN Scorecard, preference is given to studies carried out in SAC. For the purpose of this paper, the UIC data are reported for 194 WHO Member States and have been selected for each country in the following order of priority: data from the most recent known nationally representative survey carried out between 2005 and 2020 in (i) SAC, (ii) SAC and adolescents, (iii) adolescents, (iv) women of reproductive age, and (v) other adults (excluding pregnant or lactating women). In the absence of recent national surveys, sub-national data are used in the same order of priority.

In the scorecard, adequate iodine intake in SAC, women of reproductive age and other adults is defined based on a mUIC in the range 100–299 μg/L (13). Although the WHO defines adequate iodine intake in adults as a mUIC value ≥ 100 μg/L, the scientific basis for this threshold is weak (11). Thus, the national classification of iodine status using this threshold in adults should be interpreted with caution.

### Countries with adequate iodine nutrition

Cross-sectional UIC studies have been conducted in 152 out of 194 countries in the past 15 years. In 132 countries, the studies were nationally representative. The iodine intake in the general population is adequate in 118 countries (Fig. 1). The number of countries with adequate iodine intake has nearly doubled over the past 20 years from 67 in 2003 (18), to 105 in 2011 (19) and to 118 in 2020, reflecting the effectiveness of the successful implementation of salt iodization worldwide. The iodine intake in SAC is adequate in the majority of countries where salt is fortified with >15 ppm of iodine. Recent data show that mandatory salt iodization at 25 ppm ensures adequate iodine intake in all population groups, including pregnant and lactating women, who have increased requirements (20). Bouillon cubes containing iodized salt, cow’s milk and dairy products are additional important dietary iodine sources, and contribute to adequate iodine intake in many countries (21, 22).

### Countries that are iodine-deficient

**Figure 2:**
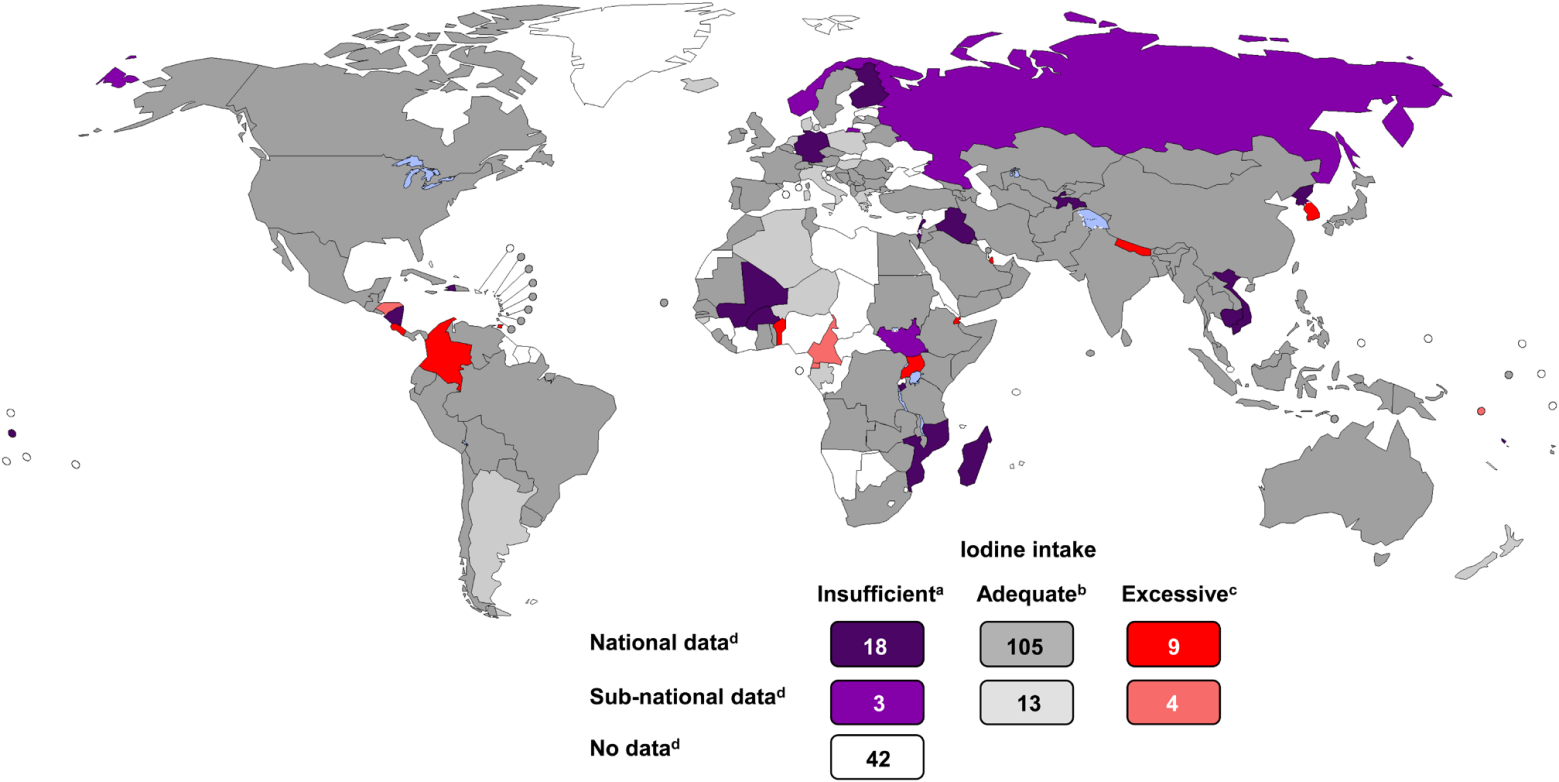
Estimated iodine nutrition in 194 WHO Member States in 2020 based on national median UIC in school-age children obtained from studies conducted between 2005–2020. ^a^Median UIC: <100 µg/L; ^b^Median UIC: 100-299 µg/L; ^c^Median UIC: ≥300 µg/L; ^d^Number of countries. Reproduced with permission from (7). UIC, Urinary iodine concentration.

In 2020, globally, 21 countries still have insufficient iodine in their diets (Fig. 2). Countries with deficient iodine intake, ranked by increasing mUIC (µg/L) are shown in Table 2. Iodine deficiency remains in all regions worldwide and affects populations at all stages of economic development. Iodized salt is available in all iodine-deficient countries, but the coverage is poor or incomplete. The iodine intake is lowest in Madagascar, where the mandatory salt iodization program fell apart due to political instability. In Vietnam, the weakening of the previous mandatory legislation allowed the introduction of non-iodized salt and iodine status decreased. In Cambodia, the production of iodized salt declined when UNICEF stopped supplying potassium iodate and the amount of iodine in fortified salt decreased. Several countries have incomplete nationwide coverage and large regional variations in iodine status, for example, Sudan, Burkina Faso and Russia. In Haiti and Iraq, natural disasters and war, respectively, disrupted the implementation and monitoring of salt production and the distribution chain. The iodine intake is also inadequate in several countries with strong health systems and otherwise successful public health programs (Norway, Germany and Finland). In Norway, iodized salt is not widely implemented and the allowed level of fortification is only 5 ppm, below the recommended minimum level of 15 ppm. Fish and seafood were assumed to provide adequate iodine intake in the population, but their iodine content is not high enough unless consumed every day, and their consumption is declining. In Germany, a major challenge is the low use of iodized salt in the production of processed foods, which contributes to most dietary salt. Finland had an effective salt iodization program for decades, but decreased consumption of iodized salt and milk resulted in lower iodine intakes. Actions to strengthen the coverage of iodized salt were recently recommended by the Finnish National Nutrition Council.

**Table 2:** Countries with deficient iodine intake in 2020, ranked by increasing median UIC (µg/L). Adapted with permission from (7)^a^.

**Country or territory**	**Median UIC (µg/L)**	**Date of survey**	**Data type**	**Study population**
Madagascar	46	2015	National	WRA
Cambodia	63	2014	National	WRA
Lebanon	66	2013	National	SAC
Mali	69	2005	National	SAC
Norway	75	2017–18	Sub-national	WRA
Tajikistan	75	2016	National	WRA
Haiti	77	2018	National	WRA
Vanuatu	77	2017	National	WRA
Burundi	80	2018	National	WRA
Israel	83	2016	National	SAC
Iraq	84	2011–12	National	SAC
Vietnam	84	2013–14	National	SAC
Samoa	88	2013	National	WRA
Germany	89	2014–17	National	SAC, Adolescents
Nicaragua	90	2018	National	WRA
South Sudan	94	2006	Sub-national	SAC
Finland	96	2017	National	Adults
Korea, DPR	97	2009–10	National	SAC
Mozambique	97	2011–12	National	WRA
Burkina Faso	99	2014	National	SAC
Russian Federation	<100	2008–20	Sub-national	SAC

^a^References for the country data available on the IGN Global Scorecard of iodine nutrition (7).

SAC, school-aged children; UIC, urinary iodine concentration; WRA, women of reproductive age.

### Countries with excessive iodine intake

Excessive intake of iodine should be avoided, particularly in previously iodine-deficient areas, since a rapid increase in iodine intake may precipitate a transient increase in hyperthyroidism (14). Conversely, the gradual introduction of iodine to deficient populations can substantially reduce hyperthyroidism due to thyroid autonomy; for example, salt iodization has halved the risk of thyrotoxicosis in Denmark (23, 24). Globally, 13 countries have documented excessive iodine in their diets, as defined by a mUIC >300 μg/L. These countries, ranked by increasing mUIC, are shown in Table 3.

**Table 3:** Countries with excessive iodine intake in 2020, ranked by increasing median UIC (µg/L). Adapted with permission from (7).^a^.

Country or territory	Median UIC (µg/L)	Date of survey	Data type	Population surveyed
Cameroon	>300	2014–2018	Sub-national	SAC
Trinidad and Tobago	311	2018	National	SAC
Costa Rica	314	2008–09	National	SAC
Nepal	314	2016	National	SAC
Benin	318	2011	National	SAC
Solomon Islands	328	2007–10	Sub-national	SAC
Djibouti	335	2015	National	SAC
Qatar	341	2014	National	SAC
Honduras	356	2005	Sub-national	SAC
Colombia	407	2015–16	National	SAC
Korea, Republic of Korea	449	2013–15	National	SAC, Adolescents
Uganda	464	2005	National	SAC
Equatorial Guinea	564	2007	Sub-national	SAC

^a^References for the country data available on the IGN Global Scorecard of iodine nutrition (7).

SAC, school-aged children; UIC, urinary iodine concentration.

Excess iodine intakes in populations can result from diets that are naturally high in iodine and/or groundwater. In South Korea, the mUIC in SAC and adolescents is 449 µg/L, mainly due to high intakes of iodine-rich seaweed (25). Djibouti, in the Horn of Africa, has excess iodine intakes due to very high iodine in groundwater. The median iodine concentration in drinking water is 92 μg/L (IQR: 37–158 μg/L) and the mUIC is 335 μg/L in SAC and 265 μg/L in pregnant women, despite only 1.6% of Djibouti salt samples being adequately iodized (>15 ppm) (26). The iodine concentration in drinking water is high also in Somalia, with a median concentration of 59 μg/L (IQR: 12–114 μg/L). Despite the low use of iodized salt (7%), the mUIC in women of reproductive age is 261 μg/L (369 μg/L in pregnant women), suggesting overall high iodine intake (27).

Iodine excess can also occur when the level of iodine added to salt is too high considering per capita salt intakes. Salt iodine levels are higher than the recommended 15–40 ppm in several countries that have national mUICs indicating iodine excess. In Cameroon, the iodine level stipulated in the national standard is high, of 100 ppm, and the mUIC is >300 µg/L in SAC. Similarly, in Honduras and Columbia, the iodine levels in the national standards are 83 and 75 ppm, respectively, and the mUICs in SAC these countries are 356 and 407 µg/L, respectively, suggesting iodine excess. In some tropical countries, iodine levels in salt were set high anticipating high losses of iodine from salt from production to the consumer; a study in Ethiopia reported a mean 57% decrease in household iodine content from an initial production level of ≈60 ppm (28). Many factors, including salt moisture and impurities, ambient humidity, light, heat, and the form (potassium iodide or iodate) in which the iodine is present, affect iodine stability in salt. However, properly packaged, good-quality iodized salt, iodized with potassium iodate, loses minimal iodine even during prolonged storage. Currently, the WHO recommends setting the salt iodization level anticipating 30% losses from production to consumption, and based on estimated per capita salt intakes. In populations where the average salt intake per capita is 5 and 10 g/day, salt should be iodized at 40 and 20 ppm, respectively (29). However, many countries have opted for higher iodization levels. In 34% of countries worldwide the legislative standard for iodine in salt is set above 40 ppm; 30 out of 137 countries with mandatory or voluntary standards for iodine in salt have standards between 41 and 59 ppm and 17 countries have a standard ≈60ppm (6).

In situations where monitoring of salt iodization programs suggests excess intakes, the salt iodization level should be reduced to bring the population mUIC <300 µg/L. A good example is Brazil. In response to surveys in 1994–95 showing a 24% goiter rate in SAC, the iodine content in salt was increased to 40–100 ppm in 1998–1999 (30). As a result, in 2000, the mUIC in SAC was 360 μg/L, and the prevalence of goiter had fallen to 1.4%. In the most recent national survey, which began in 2008–2009 and was completed in 2013–2014, the national mUIC was 277 µg/L, ranging from 248 µg/L in the South to 299 µg/L in the Northeast. In response, the fortification level of iodine in salt has been reduced to 15–45 mg (30). These Brazilian data highlight the value of regular monitoring of both salt iodization programs and of population iodine status.

### Fragility and durability of iodized salt programs

In countries that have recently introduced iodized salt programs, sustainability has become a major focus. These programs are fragile, and IDD in children can rapidly return if the iodized salt supply is disrupted (31). Recent countries where programs have slipped are Vietnam and Cambodia (7). Salt iodization programs require a long-term commitment from governments and the salt industry (32).

On the other hand, entrenched iodine programs are often surprisingly durable, even during national crises. Since 2014, Yemen has been devastated by civil war disrupting food supplies and causing widespread acute malnutrition (33). Remarkably, the most recent iodine survey reported that iodine status remains adequate in SAC with a mUIC of 101 μg/L (7). UNICEF estimates that 49% of households had access to iodized salt at the beginning of the crisis (34). Decades of conflict in Afghanistan have caused a chronic humanitarian crisis with high rates of severe acute malnutrition, stunting and anemia (35). In the war-torn country, household coverage with iodized salt has remained at ≈55% (34), and the latest national iodine survey reported iodine sufficiency in SAC, with a mUIC of 171 μg/L (7).

**Iodized salt programs succeed in many countries with the highest burden of malnutrition**

Iodized salt programs have been successful in countries with otherwise chronic and severe undernutrition. Of the 15 countries with the highest rates of stunting (34), in which more than 38% of preschool children are stunted from chronic undernutrition, 10 are iodine-sufficient at the national level (Afghanistan, Chad, Democratic Republic of Congo, Guatemala, Malawi, Niger, Papua New Guinea, Sudan, Timor-Leste, Yemen). Only 3 are iodine-deficient at the national level (Burundi, Mozambique and Madagascar) while the Central African Republic and Eritrea have no recent UIC data.

Nearly half of the world’s stunted children (144 million children) live in just three countries: India, Nigeria and Pakistan (34). Despite having unstable food systems that cannot provide adequate nutrition to millions of children, all three countries are iodine-sufficient at the national level and have effective iodized salt programs. Population coverage with iodized salt is a remarkable 93% in India and Nigeria, and 69% in Pakistan. The latest national nutrition survey in Pakistan in 2011 reported little progress on core maternal and childhood nutrition indicators and no gains in reducing most micronutrient deficiencies (36). But one bright spot was the striking improvement in iodine status in children and women; between 2001 and 2011 the mUIC increased from 89 to 124 μg/L in SAC , and from 63 to 105 μg/L in women of reproductive age (36).

### Equity of iodine programs

Around the world, iodine programs are reaching the poorest of the poor. Of the 15 poorest countries in the world, with per capita GDPs of <700 USD (37), 10 are iodine-sufficient at the national level (Afghanistan, Democratic Republic of Congo, Guinea Bissau, Liberia, Malawi, Niger, Sierra Leone, Somalia, Sudan and Togo), 3 are mild-to-moderately deficient (Burundi, Mozambique and Madagascar) and the Central African Republic and Eritrea have no recent data.

Moreover, half of the world’s 736 million extremely poor people live in just five countries: India, Nigeria, the Democratic Republic of Congo, Ethiopia and Bangladesh. All five countries are iodine-sufficient at the national level, with high household coverage with iodized salt (HHIS): India (93% HHIS, mUIC in women of reproductive age 178 μg/L), Nigeria (93% HHIS, mUIC in SAC 130 μg/L), Democratic Republic of Congo (82% HHIS, mUIC in SAC 249 μg/L), Ethiopia (86% HHIS, mUIC in SAC 104 μg/L), and Bangladesh (68% HHIS, mUIC in SAC 146 μg/L).

Equity of iodized salt programs has also been assessed within countries. For iodized salt programs to be effective, they need to reach the poorest households in countries. According to UNICEF, out of the 77 countries with equity data on iodized salt, only 40% show equity between the richest and poorest households for consumption of iodized salt; in the remaining countries (58%), the richest had coverage estimates that are at least 10% higher than the poorest (34). Countries with higher overall coverage tend to have a more equitable distribution of coverage.

## Conclusions and future perspectives

There has been remarkable progress toward increasing iodine intakes and eliminating IDD over the past three decades. Since 1990, the number of iodine-deficient countries has fallen from 113 (38) to 21 (7). This is mainly due to the scale-up of salt iodization in most countries. However, to consolidate and sustain these achievements, and to reach the remaining iodine-deficient countries, refinements in salt iodization programs are required.

The original concept of salt iodization was for it to be universal (i.e. the iodization of all food-grade salt used in households and food processing) (8). However, many national programs have focused mainly on household salt being adequately iodized. The consumption of salt through processed foods is increasing in many countries, and in many middle- and high-income countries most salt is consumed through processed foods (39). If the salt contained in such foods is well-iodized, it can be an important source of iodine (40). National salt iodization programs should encourage and monitor the use of iodized salt in processed foods. Iodine coming from processed foods may help explain why the mUIC is sufficient in populations where household iodized salt coverage is low (18).

There is growing worldwide interest in reducing salt intake to prevent hypertension and other non-communicable diseases. Concerns have been raised that programs to reduce dietary salt could adversely impact programs to prevent iodine deficiency, and, conversely, that iodizing salt could encourage higher salt intakes (41). The WHO has emphasized that these programs are compatible but need to be carefully aligned (29). Both programs have common elements, including program monitoring via urine collections and interactions with the food and salt industries. This provides an opportunity to share and leverage resources and approaches to be more effective and efficient (29). As salt intakes fall, iodine levels can be increased in salt to adjust for the recommended reduction in dietary salt to less than 5 g/day (8).

National level mUIC may hide disparities in iodine intake among sub-groups, including those in a specific geographic region, lower socioeconomic status, with varying diets and/or salt sources. If resources allow, the iodine intakes should be assessed among different subsets of the population, particularly among groups vulnerable to deficiency, such as the poor in remote areas and those obtaining salt from small-scale salt producers who may not iodize their salt. Such stratified analyses may help identify remaining sub-national challenges and allow refinements to improve programs.

UIC surveys are traditionally done in children, because they are a convenient population, easy to reach through school-based surveys and usually representative of the general population (2). Recently, greater emphasis has been placed on surveying women of reproductive age and pregnant women (9). Pregnant women have sharply higher iodine requirements, and are a key target group of iodine programs. More data is becoming available, suggesting that many pregnant women in both low- and high-income countries, including the United States of America (42) and several European countries (43), have low iodine intakes. However, the mUIC thresholds that indicate iodine deficiency in pregnant and nonpregnant women remain uncertain. More research is needed on evidence-based thresholds for mUIC in these two populations to define iodine status.

## Declaration of interest

The authors are members of the Iodine Global Network, a non-profit, non-governmental organization based in Ottawa, Ontario, Canada, but declare no further conflicts of interest.

## Funding

This research did not receive any specific grant from any funding agency in the public, commercial or not-for-profit sector.

## References

[1] Zimmermann MB Iodine deficiency. Endocrine Reviews 2009 30 376–408. (10.1210/er.2009-0011) 19460960

[2] WHO, UNICEF & ICCIDD. Assessment of Iodine Deficiency Disorders and Monitoring their Elimination: A Guide for Programme Managers. Geneva: World Health Organization, 2007.

[3] Kelly FC Snedden WW. Prevalence and geographical distribution of endemic goiter. Monograph Series: World Health Organization. Geneva: World Health Organization, 1960. 13752376

[4] Walker SP Wachs TD Gardner JM Lozoff B Wasserman GA Pollitt E Carter JA & International Child Development Steering Group. Child development: risk factors for adverse outcomes in developing countries. Lancet 2007 369 145–157. (10.1016/S0140-6736(0760076-2) 17223478

[5] WHO. WHO e-Library of Evidence for Nutrition Actions (eLENA), 2021. (Available at: https://www.who.int/elena/en/). Accessed on 26 Jan 2021.

[6] Global fortification data exchange (GFDx), 2021. (Available at: http://www.fortificationdata.org). Accessed on 11 Feb 2021

[7] Iodine Global Network. Global Scorecard of Iodine Nutrition in 2020 in the General Population Based on Schoolage Children. Ottawa, Canada: IGN, 2021. (Available at: https://www.ign.org/scorecard.htm). Accessed on 7 May 2021.

[8] WHO. Fortification of Food-Grade Salt with Iodine for the Prevention and Control of Iodine Deficiency Disorders. WHO Guidelines Approved by the Guidelines Review Committee. Geneva: World Health Organization, 2014. 25473709

[9] UNICEF. Guidance on the Monitoring of Salt Iodization Programmes and Determination of Population Iodine Status. New York: United Nations Children's Fund, 2018.

[10] UNICEF. United Nations Children's Fund, Division of data, analysis, planning and monitoring. UNICEF Global Databases on Iodized salt, New York, 2019. (Available at: https://data.unicef.org/wp-content/uploads/2017/12/Global-Databases-on-Iodine.xlsx11). Accessed on 11 February 2021.

[11] Zimmermann MB Andersson M. Assessment of iodine nutrition in populations: past, present, and future. Nutrition Reviews 2012 70 553–570. (10.1111/j.1753-4887.2012.00528.x) 23035804

[12] König F Andersson M Hotz K Aeberli I Zimmermann MB Ten repeat collections for urinary iodine from spot samples or 24-h samples are needed to reliably estimate individual iodine status in women. Journal of Nutrition 2011 141 2049–2054. (10.3945/jn.111.144071) 21918061

[13] Zimmermann MB Aeberli I Andersson M Assey V Yorg JA Jooste P Jukić T Kartono D Kusić Z Pretell E ***et al***. Thyroglobulin is a sensitive measure of both deficient and excess iodine intakes in children and indicates no adverse effects on thyroid function in the UIC range of 100–299 mug/L: a UNICEF/ICCIDD study group report. Journal of Clinical Endocrinology and Metabolism 2013 98 1271–1280. (10.1210/jc.2012-3952) 23345097

[14] Zimmermann MB Boelaert K. Iodine deficiency and thyroid disorders. Lancet: Diabetes and Endocrinology 2015 3 286–295. (10.1016/S2213-8587(1470225-6) 25591468

[15] Andersson M de Benoist B Delange F Zupan J WHO Secretariat on behalf of the participants to the consultation. Prevention and control of iodine deficiency in pregnant and lactating women and in children less than 2-years-old: conclusions and recommendations of the Technical Consultation. Public Health Nutrition 2007 10 1606–1611. (doi: 10.1017/S1368980007361004) 10.1017/S1368980007361004 18053287

[16] Stinca S Andersson M Weibel S Herter-Aeberli I Fingerhut R Gowachirapant S Hess SY Jaiswal N Jukic T Kusic Z Dried blood spot thyroglobulin as a biomarker of iodine status in pregnant women. Journal of Clinical Endocrinology and Metabolism 2017 102 23–32. (10.1210/jc.2016-2829) 27732337

[17] Dold S Zimmermann MB Aboussad A Cherkaoui M Jia QZ Jukic T Kusic Z Quirino A Sang Z Luis TOL ***et al***. Breast milk iodine concentration is a more accurate biomarker of iodine status than urinary iodine concentration in exclusively breastfeeding women. Journal of Nutrition 2017 147 528–537. (10.3945/jn.116.242560) 28228508

[18] Andersson M Takkouche B Egli I Allen HE de Benoist B Current global iodine status and progress over the last decade towards the elimination of iodine deficiency. Bulletin of the World Health Organization 2005 83 518–525. 16175826 PMC2626287

[19] Andersson M Karumbunathan V Zimmermann MB Global iodine status in 2011 and trends over the past decade. Journal of Nutrition 2012 142 744–750. (10.3945/jn.111.149393) 22378324

[20] Dold S Zimmermann MB Jukic T Kusic Z Jia Q Sang Z Quirino A San Luis TOL Fingerhut R Kupka R Universal salt iodization provides sufficient dietary iodine to achieve adequate iodine nutrition during the first 1000 days: a cross-sectional multicenter study. Journal of Nutrition 2018 148 587–598. (10.1093/jn/nxy015) 29659964

[21] Abizari AR Dold S Kupka R Zimmermann MB More than two-thirds of dietary iodine in children in northern Ghana is obtained from bouillon cubes containing iodized salt. Public Health Nutrition 2017 20 1107–1113. (10.1017/S1368980016003098) 27903312 PMC10261352

[22] van der Reijden OL Zimmermann MB Galetti V Iodine in dairy milk: sources, concentrations and importance to human health. Best Practice and Research: Clinical Endocrinology and Metabolism 2017 31 385–395. (10.1016/j.beem.2017.10.004) 29221567

[23] Petersen M Bulow Pedersen I Knudsen N Andersen S Jorgensen T Perrild H Ovesen L, Banke Rasmussen L Thuesen BH Carlé A Changes in subtypes of overt thyrotoxicosis and hypothyroidism following iodine fortification. Clinical Endocrinology 2019 91 652–659. (10.1111/cen.14072) 31400012

[24] Zimmermann MB Salt iodization halves risk of thyrotoxicosis in Denmark. Nature Reviews. Endocrinology 2019 15 632–633. (10.1038/s41574-019-0261-z) 31492922

[25] Kang MJ Hwang IT Chung HR Excessive iodine intake and subclinical hypothyroidism in children and adolescents aged 6–19 years: results of the sixth korean national health and nutrition examination survey, 2013–2015. Thyroid 2018 28 773–779. (10.1089/thy.2017.0507) 29737233

[26] Farebrother J Zimmermann MB Abdallah F Assey V Fingerhut R Gichohi-Wainaina WN Hussein I Makokha A Sagno K Untoro J Effect of excess iodine intake from iodized salt and/or groundwater iodine on thyroid function in nonpregnant and pregnant women, infants, and children: a multicenter study in east africa. Thyroid 2018 28 1198–1210. (10.1089/thy.2018.0234) 30019625

[27] Ministry of Health, The Federal Government of Somalia, Federal Member States, Somaliland, UNICEF, Brandpro, GroundWork. Somalia Micronutrient Survey 2019. Ministry of Health, Mogadishu, Somalia, 2020.

[28] Shawel D Hagos S Lachat CK Kimanya ME Kolsteren P Post-production losses in iodine concentration of salt hamper the control of iodine deficiency disorders: a case study in northern ethiopia. Journal of Health, Population, and Nutrition 2010 28 238–244. (10.3329/jhpn.v28i3.5550) PMC2980888 20635634

[29] WHO. Salt Reduction and Iodine Fortification Strategies in Public Health: Report of a Joint Technical Meeting. Geneva: World Health Organization, 2014.

[30] Cesar AJ Santos SI, Black ER Chrestani DMA, Duarte AF Nilson FEA. Iodine status of Brazilian school-age children: a national cross-sectional survey. Nutrients 2020 12 1077. (10.3390/nu12041077) PMC7230565 32295049

[31] Zimmermann MB Wegmuller R Zeder C Torresani T Chaouki N Rapid relapse of thyroid dysfunction and goiter in school-age children after discontinuation of salt iodization. American Journal of Clinical Nutrition 2004 79 642–645. (10.1093/ajcn/79.4.642) 15051609

[32] Dunn JT Complacency: the most dangerous enemy in the war against iodine deficiency. Thyroid 2000 10 681–683. (10.1089/10507250050137752) 11014312

[33] FAO, UNICEF, WFP & WHO. Yemen - Acute Malnutrition Hits Record Levels in Yemen with a Devestating Toll on Children under Five. IPC Acute Malnutrition Analysis January 2020-March 2021. Yemen: WFP & world health organization, 2021. (Available at: http://www.ipcinfo.org/fileadmin/user_upload/ipcinfo/docs/IPC_Yemen_Acute_Malnutrition_2020Jan2021Mar.pdf). Accessed on 15 February 2021.

[34] UNICEF, WHO, World Bank. UNICEF/WHO/World Bank Joint Child Malnutrition Estimates, 2020, 2020, ed. New York: United Nations Children's Fund, 2021. (Available at: https://data.unicef.org/topic/nutrition/malnutrition/). Accessed on 26 Jan 2021.

[35] UNICEF. Malnutrition. New York: United Nations Children's Fund 2021(Available at: https://www.unicef.org/afghanistan/topics/malnutrition). Accessed on 26 Jan 2021.

[36] Aga Khan University & Pakistan Medical Research Council. Nutrition Wing Ministry of Health, UNICEF Pakistan. Pakistan National Nutrition Survey 2011. Karachi: Aga Khan University, 2011.

[37] The World Bank. World Bank data Catalog 2021. (Available at: https://data.worldbank.org/). Accessed on 26 Jan 2021.

[38] WHO, UNICEF & ICCIDD. Global Prevalence of Iodine Deficiency Disorders Micronutrient Deficiency Information System Working Paper 1. Geneva: World Health Organizaion, 1993.

[39] Bhat S Marklund M Henry ME Appel LJ Croft KD Neal B Wu JHY A systematic review of the sources of dietary salt around the world. Advances in Nutrition 2020 11 677–686. (10.1093/advances/nmz134) 31904809 PMC7231587

[40] Spohrer R Garrett GS Timmer A Sankar R Kar B Rasool F Locatelli-Rossi L Processed foods as an integral part of universal salt iodization programs: a review of global experience and analyses of Bangladesh and Pakistan. Food and Nutrition Bulletin 2012 33 S272–S280. (10.1177/15648265120334S303) 23444708

[41] Campbell N Dary O Cappuccio FP Neufeld LM Harding KB Zimmermann MB Collaboration to optimize dietary intakes of salt and iodine: a critical but overlooked public health issue. Bulletin of the World Health Organization 2012 90 73–74. (10.2471/BLT.11.092080) 22271970 PMC3260577

[42] Caldwell KL Pan Y Mortensen ME Makhmudov A Merrill L Moye J Iodine status in pregnant women in the national children's study and in U.S. women (15–44 years), national health and nutrition examination survey 2005–2010. Thyroid 2013 23 927–937. (10.1089/thy.2013.0012) 23488982 PMC3752509

[43] Zimmermann MB Gizak M Abbott K Andersson M Lazarus JH Iodine deficiency in pregnant women in Europe. Lancet: Diabetes and Endocrinology 2015 3 672–674. (10.1016/S2213-8587(1500263-6) 26268907

